# Insuficiência Cardíaca com Fração de Ejeção Ventricular Esquerda Supranormal - Estado da Arte

**DOI:** 10.36660/abc.20190835

**Published:** 2021-05-06

**Authors:** Ziyin Huang, Yufeng Jiang, Yafeng Zhou

**Affiliations:** 1 First Affiliated Hospital of Soochow University Jiangsu Province China First Affiliated Hospital of Soochow University, Jiangsu Province – China; 2 Dushu Lake Hospital Affiliated to Soochow University Jiangsu Province China Dushu Lake Hospital Affiliated to Soochow University, Jiangsu Province – China

**Keywords:** Insuficiência Cardíaca, Fração de Ejeção Ventricular, Insuficiência Cardíaca Diastólica, Mortalidade, Cardiomegalia, Ecocardiografia/métodos, Prognóstico

## Abstract

Em 2019, um artigo publicado no *European Heart Journal* reconheceu pela primeira vez a insuficiência cardíaca (IC) com fração de ejeção do ventrículo esquerdo (FEVE) ≥ 65% como um novo fenótipo de IC, ou a insuficiência cardíaca com fração de ejeção supranormal (ICFEsn), com o objetivo principal de promover a investigação desta nova categoria. Eles analisaram a mortalidade em pessoas com IC e descobriram que havia uma relação em forma de U entre a mortalidade e a FEVE. Sendo assim, os pacientes com ICFEsn tinham uma mortalidade geral mais alta em comparação com outros pacientes diagnosticados com IC com fração de ejeção preservada (ICFEp). Este artigo descreve a situação atual da ICFEsn e discute as perspectivas futuras com base nos resultados preliminares de nosso grupo. Para melhor tratar os pacientes com ICFEsn, é fundamental que cardiologistas e médicos entendam as diferenças e semelhanças desse novo fenótipo.

## Introdução

Estima-se que mais de 100 milhões de pessoas sofram de insuficiência cardíaca (IC) em todo o mundo.^1^ No estudo DIGITALIS realizado no Brasil, 64,2% desses pacientes foram diagnosticados com IC com fração de ejeção preservada (ICFEp). Recentemente, um novo tipo de IC, denominado IC com fração de ejeção de faixa média (ICFEfm), foi descrito. De acordo com dados não publicados da base de dados DIGITALIS, a prevalência de IC com fração de ejeção reduzida (ICFEr) foi de 19%, a ICFEfm foi de 22% e a ICFEp foi de 59%. Isso mostra que a ICFEp é responsável por uma grande proporção de casos de IC.^2^^–^^4^

Em um artigo publicado no *European Heart Journal*, que investigou a relação entre a fração de ejeção do ventrículo esquerdo (FEVE) avaliada clinicamente e a mortalidade em uma grande coorte clínica, foi encontrada uma relação em forma de U entre a mortalidade e a FEVE, sugerindo que pode ser inadequado reunir todos os pacientes com ICFEp em um único grupo.^5^ Esses resultados podem anunciar o reconhecimento de um novo fenótipo de IC com FEVE ≥ 65%, que é caracterizado por uma mortalidade por todas as causas mais elevada.^6^

A insuficiência cardíaca com fração de ejeção supranormal (ICFEsn) demonstrou ter manifestações clínicas, tratamento e prognóstico especiais. Mais pesquisas precisam ser realizadas para explorar as características e o tratamento dessa nova categoria de IC. O fenótipo ICFEsn pode ser reconhecido como uma classificação clinicamente relevante por diretrizes nacionais e internacionais.

### Fisiopatologia e Patologia da ICFEsn

O desempenho ideal do ventrículo esquerdo (VE) depende de duas condições: um VE complacente, que permite seu preenchimento a partir da pressão atrial esquerda baixa durante a diástole, e um VE firme na sístole, que ejeta o volume sistólico à pressão arterial. O ecocardiograma é a técnica de imagem mais utilizada para avaliar a função diastólica e sistólica, e a FEVE é o índice mais utilizado. Pacientes com diagnóstico de ICFEp frequentemente apresentam FEVE normal (FEVE≥50%) e que é caracterizada por disfunção diastólica.

**Figura 1:**
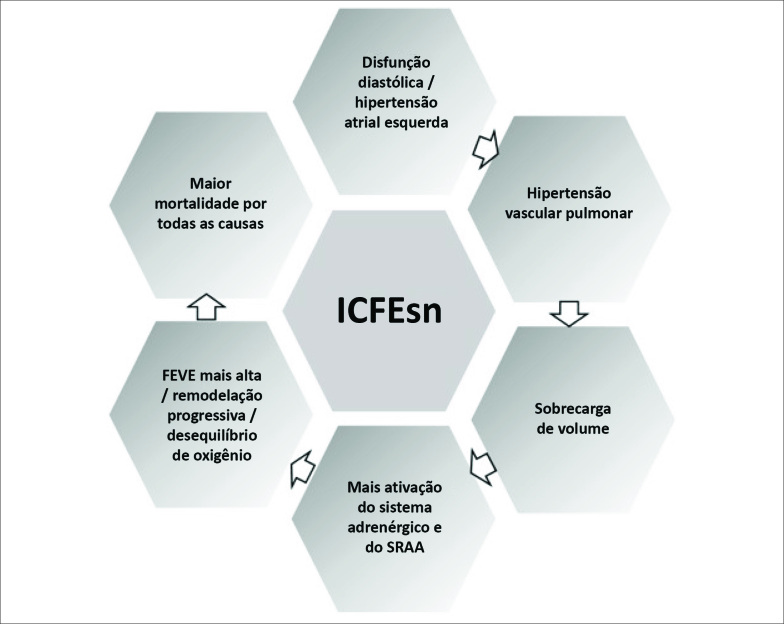
Mecanismos da ICFEsn. ICFEsn: insuficiência cardíaca com fração de ejeção supranormal; FEVE: fração de ejeção do ventrículo esquerdo; SRAA: Sistema renina-angiotensina-aldosterona

Como um tipo especial de ICFEp, a ICFEsn também é caracterizada por disfunção diastólica. Em um estudo publicado recentemente, concluiu-se que pacientes com FEVE mais alta têm um prognóstico pior.^5^ A possível razão para isso é que pessoas com coração hipertrófico (e FEVE muito alta) podem bombear maior volume de sangue a cada batimento e serem particularmente suscetíveis à isquemia mediada pelo suprimento de oxigênio.^7^ A ativação neuro-hormonal pode ser outra razão para o mau prognóstico na ICFEsn. A FEVE mais alta pode ser devida à maior ativação do sistema adrenérgico e do sistema renina-angiotensina-aldosterona (SRAA) e a maior ativação desses sistemas pode contribuir para a remodelação cardíaca progressiva e disfunção contrátil.^8^ Pacientes com coração remodelado tinham maior probabilidade de sofrer parada cardíaca ou fibrilação ventricular, quando comparados ao grupo com FE normal.^9^ Os motivos descritos acima podem explicar o aumento da mortalidade dos pacientes com ICFEsn (Figura 1).

### Abordagem diagnóstica

De acordo com as últimas Diretrizes da ESC para IC aguda e crônica, o diagnóstico de IC é baseado na combinação de sintomas, sinais, peptídeos natriuréticos e resultados do ecocardiograma.^1^ Em uma análise recente de um grande conjunto de dados, os pesquisadores começaram a definir pacientes com FEVE ≥65% como um novo tipo de HF, denominado ICFEsn. Como um tipo especial de IC diastólica, o diagnóstico de ICFEsn pode exigir a presença de sinais ou sintomas de IC, níveis elevados de BNP, evidência de função sistólica normal do VE e evidência de disfunção diastólica ou marcadores substitutos que incluem hipertrofia do VE, aumento do AE e fibrilação atrial.^10^ Ao mesmo tempo, a FEVE ≥65% medida pelo ecocardiograma é uma das condições essenciais para o diagnóstico de ICFEsn. Os critérios clínicos detalhados para o diagnóstico de ICFEsn são apresentados na tabela 1.

**Tabela 1:** Critérios clínicos na investigação de ICFEsn

Categorias	Critérios
Sintomas e/ou sinais de IC	Falta de ar, dispneia paroxística noturna, Tolerância reduzida ao exercício, Fadiga, cansaço, tempo aumentado para recuperação após o exercício, Edema de tornozelo
	Pressão venosa jugular elevada, refluxo hepatojugular, Terceira bulha cardíaca (ritmo de galope), impulso apical deslocado lateralmente
FEVE	FEVE ≥65%
Níveis elevados de PNs	BNP>35 pg/mL and/ou NT-proBNP>125 pg/mL
Evidência objetiva de outras alterações funcionais e estruturais cardíacas subjacentes à IC	índice de volume atrial esquerdo (IVAE), índice de massa ventricular esquerda (IMVE), E /e', e' septal e parede lateral média, *strain* longitudinal ou velocidade de regurgitação tricúspide (VRT)
Um teste de estresse ou pressão de enchimento do VE elevada medida invasivamente	Um teste de estresse diastólico realizado com ecocardiografia, pressão capilar pulmonar em cunha (PCPC), pressão diastólica final do ventrículo esquerdo (PDFVE)

IC: insuficiência cardíaca; FEVE: fração de ejeção do ventrículo esquerdo.

### Tratamento da ICFEsn

Embora já exista uma classificação rudimentar de IC usada para o tratamento de precisão na IC, uma verdadeira abordagem da Medicina de Precisão para a IC ainda está em sua infância, e o tratamento de pacientes com ICFEp e ICFEr também é baseado em uma abordagem modelo “tamanho único”.

Com base na patologia e fisiopatologia da ICFEsn, pode-se deduzir que os pacientes com ICFEsn podem ser sensíveis a vários medicamentos tradicionais que são benéficos para outros tipos de IC. Entretanto, nenhum medicamento mostrou quaisquer benefícios confirmados experimentalmente. Por exemplo, os β-bloqueadores podem ser úteis para o tratamento de ICFEsn, pois seu efeito cronotrópico negativo (diminuição da frequência cardíaca) aumenta o período de enchimento diastólico e o suprimento de oxigênio para o miocárdio. IECA, BRA e espironolactona também podem ter um efeito na ICFEsn, diminuindo a remodelação progressiva. Mas todos os medicamentos precisam de estudos prospectivos e testes clínicos para identificar seus efeitos.

O treinamento físico em pacientes com ICFEsn pode beneficiar os pacientes, melhorando a tolerância ao exercício e controlando a obesidade. Mas a quantidade certa de exercício para ICFEsn requer ensaios clínicos para confirmá-la. Em artigo publicado recentemente, pesquisadores avaliaram o tema Medicina de Precisão em ICFEp. A Medicina de Precisão fornece um novo conceito para o tratamento da IC e pode também ter um efeito na ICFEsn.^11^

### Perspectivas futuras

**Figura 2:**
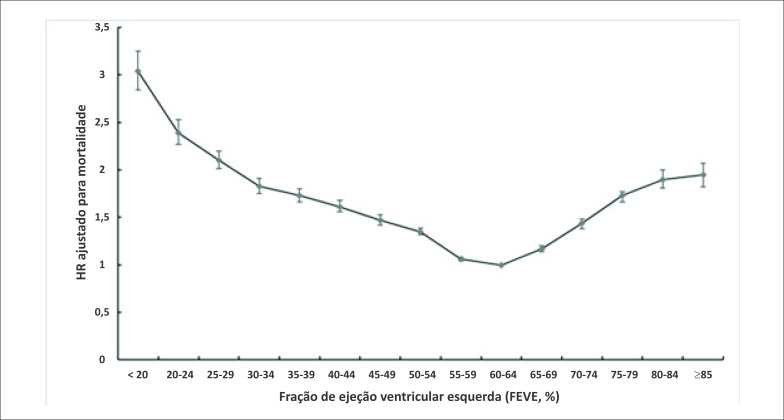
Hazard Ratio ajustado para mortalidade de acordo com a FEVE.

Nosso estudo recente calculou as taxas de risco (*hazard ratios*, HRs) ajustadas para mortalidade com um valor mais baixo de FEVE de 60-64% e descobriu que o desvio da FEVE de 60% a 64% foi associado a uma sobrevida mais baixa (Figura 2). Pacientes com ICFEsn tiveram um risco de morte quase 2 vezes maior do que pacientes com FEVE entre 60%-64%. Também dividimos os pacientes com ICFEsn em dois grupos, tratando-os com IECA / BRA ou não tratando. Os dados preliminares mostraram um efeito favorável na sobrevivência do paciente. IECA/BRA podem ser agentes terapêuticos atraentes para o tratamento de pacientes com ICFEsn. Mais estudos prospectivos e ensaios clínicos randomizados são essenciais para o estabelecimento de terapias com recomendações baseadas em evidências sólidas.

Após a proposta dessa nova categoria de IC, haverá cada vez mais pesquisas sobre esse tipo de IC, contribuindo para um melhor entendimento desse novo fenótipo, e se um aumento na mortalidade para FEVE ≥65% se aplica a pessoas com hipertensão e obesidade continua sendo uma questão significativa que merece mais estudos.

## Conclusões

Com base na pesquisa existente, concluímos que os pacientes com diagnóstico de ICFEsn (FEVE≥65%) têm uma manifestação clínica especial, que é caracterizada por uma mortalidade por todas as causas mais elevada em comparação com outros pacientes com ICFEp.
