# Supplementary material for: Insuficiência Cardíaca com Fração de Ejeção Ventricular Esquerda Supranormal - Estado da Arte
Source: Arq Bras Cardiol. 2021 May 6;116(5):1019–22. [Article in Portuguese] doi: 10.36660/abc.20190835 (PMC8121464; doi:10.36660/abc.20190835)
Supplement: Translation

# Translation: Heart Failure with Supra-normal Left Ventricular Ejection Fraction – State of the Art

**Authors:** Ziyin Huang, Yufeng Jiang, Yafeng Zhou

**Affiliations:** 1 Jiangsu Province China First Affiliated Hospital of Soochow University, Jiangsu Province – China; 2 Jiangsu Province China Dushu Lake Hospital Affiliated to Soochow University,2 Jiangsu Province – China

**Keywords:** Heart Failure, Stroke Volume, Heart Failure Diastolic, Mortality, Cardiomegaly, Echocardiography/methods, Prognosis

## Abstract

In 2019, an article published in the European Heart Journal recognized for the first time heart failure (HF) with left ventricular ejection fraction (LVEF)≥ 65% as a new HF phenotype, heart failure with supra-normal left ventricular ejection fraction (HFsnEF), with the main purpose of promoting research on this new category. They analyzed mortality in people with HF and found that there was a u-shaped relationship between mortality and LVEF. Accordingly, HFsnEF patients had a higher all-cause mortality compared with other patients diagnosed with HF with preserved ejection fraction (HFpEF). This article describes the current situation of HFsnEF and discusses future perspectives based on the preliminary results of our group. To better treat patients with HFsnEF, it is fundamental that cardiologists and physicians understand the differences and similarities of this new phenotype.

## Introduction

It is estimated that more than 100 million people suffer from heart failure (HF) worldwide.^1^ In the DIGITALIS trial performed in Brazil, 64.2% of these patients were diagnosed with HF with preserved ejection fraction (HFpEF). Recently, a new type of HF, called HF with mid-range ejection fraction (HFmrEF) has been described. According to unpublished data from the DIGITALIS database, the prevalence of HF with reduced ejection fraction (HFrEF) was 19%, HFmrEF was 22% and HFpEF was 59%. It shows that HFpEF accounts for a large proportion of HF.^2^^–^^4^

In an article published in the European Heart Journal, which investigated the relationship between clinically assessed left ventricular ejection fraction (LVEF) and mortality in a large clinical cohort, a u-shaped relationship between mortality and LVEF was found, suggesting that it may be inappropriate to pool all patients with HFpEF into a single group.^5^ These results may herald the recognition of a new phenotype of HF with LVEF ≥ 65%, which is characterized by a higher all-cause mortality.^6^

Heart failure with supra-normal ejection fraction (HFsnEF) has shown to have special clinical manifestations, treatment and prognosis. More research needs to be carried out to explore the characteristics and treatment of this new HF category. The HFsnEF phenotype might be recognized as a clinically relevant classification by national and international guidelines.

### Pathophysiology and Pathology of HFsnEF

Optimal left ventricular (LV) performance depends on two conditions: a compliant LV, which allows it to fill from low left atrial pressure during diastole and a firm LV in systole, which ejects the stroke volume at arterial pressure. The echocardiogram is the most commonly used imaging technique to evaluate diastolic and systolic function, and the LVEF is the most widely used index. Patients diagnosed with HFpEF often have a normal LVEF (LVEF≥50%) and that is characterized by diastolic dysfunction.

As a special type of HFpEF, HFsnEF is also characterized by diastolic dysfunction. In a recently published study, it was concluded that patients with higher LVEF have a poorer prognosis.^5^ The possible reason is that people with hypertrophic hearts (and very high LVEF) may pump more volume of blood with each beat and be particularly susceptible to oxygen supply–mediated ischemia.^7^ Neurohormonal activation may be another reason for poor prognosis in HFsnEF. Higher LVEF may be due to higher activation of the adrenergic and renin–angiotensin–aldosterone system (RAAS) and higher activation of these systems may contribute to progressive heart remodeling and contractile dysfunction.^8^ Patients with a remodeled heart were more likely to suffer from cardiac arrest or ventricular fibrillation, when compared with the normal EF group.^9^ The reasons described above might explain the increased mortality of the HFsnEF patients (Figure 1).

**Figure 1:** HFsnEF mechanisms. HFsnEF: heart failure with supra-normal left ventricular ejection fraction; LVEF: left ventricular ejection fraction; RAAS: Renin-angiotensin-aldosterone system.

### Diagnostic approach

According to the latest ESC Guidelines for acute and chronic HF, the diagnosis of HF is based on the combination of symptoms, signs, natriuretic peptides and results of the echocardiogram.^1^ In a recent analysis of a large dataset, researchers started defining patients with LVEF≥65% as a new type of HF, called HFsnEF. As a special type of diastolic HF, the diagnosis of HFsnEF may require the presence of signs or symptoms of HF, elevated BNP levels, evidence of normal systolic LV function and evidence of diastolic dysfunction or surrogate markers that include LV hypertrophy, LA enlargement, and atrial fibrillation.^10^ At the same time, LVEF ≥65% measured by the echocardiogram is one of the essential conditions for the diagnosis of HFsnEF. The detailed clinical criteria for the diagnosis of HFsnEF are shown in table 1.

**Table 1:** Clinical criteria in the investigation of HFsnEF

Categories	Criteria
Symptoms and/or signs of HF	Breathlessness, Paroxysmal nocturnal dyspnea, Reduced exercise tolerance, Fatigue, tiredness, increased time to recover after exercise, Ankle swelling
	Elevated jugular venous pressure, Hepatojugular reflux, Third heart sound (gallop rhythm), Laterally displaced apical impulse
LVEF	LVEF ≥65%
Elevated levels of NPs	BNP>35 pg/mL and/or NT-proBNP>125 pg/mL
Objective evidence of other cardiac functional and structural alterations underlying HF	Left atrial volume index (LAVI), left ventricular mass index (LVMI), E/e', mean e' septal and lateral wall, longitudinal strain or tricuspid regurgitation velocity (TRV)
A stress test or invasively measured elevated LV filling pressure	A diastolic stress test performed with echocardiography, pulmonary capillary wedge pressure (PCWP), left ventricular end diastolic pressure (LVEDP)

HF: heart failure; LVEF: left ventricular ejection fraction.

### Treatment of HFsnEF

Although there is already a rudimentary classification of HF used for precision treatment in HF, a true Precision Medicine approach to HF is currently still in its infancy, and the treatment of HFpEF and HFrEF patients is also based on a “one-size-fits-all” approach.

Based on the pathology and pathophysiology of HFsnEF, one can deduce that patients with HFsnEF may be sensitive to several traditional drugs that are beneficial for other kinds of HF. But no drugs have shown any experimentally confirmed benefits. For example, β-blockers may be useful for the treatment of HFsnEF, as its negative chronotropic effect (decreased heart rate) increases the diastolic filling period and oxygen supply to the myocardium. ACEIs, ARBs and spironolactone may also have an effect on HFsnEF by decreasing progressive remodeling. But all of the drugs need prospective studies and clinical trials to identify their effects.

Exercise training in patients with HFsnEF may benefit patients by improving exercise tolerance and managing obesity. But the right amount of exercise for HFsnEF requires clinical trials to confirm it. In a recently published article, researchers assessed the topic of Precision Medicine in HFpEF. Precision Medicine provides a new concept for the treatment of HF and it may also have an effect on HFsnEF.^11^

### Future perspectives

Our recent study has calculated the adjusted hazard ratios (HRs) for mortality with a nadir at LVEF of 60–64% and found that LVEF deviation from 60% to 64% was associated with poorer survival (Figure 2). HFsnEF patients had a risk of death that was almost 2-times higher than that of patients with LVEF between 60%-64%. We also divided HFsnEF patients into two groups by treating them with ACEIs/ARBs or not. Preliminary data showed a favorable effect on patient survival. ACEIs/ARBs may be attractive therapeutic agents to treat patients with HFsnEF. More prospective studies and randomized clinical trials are essential for the establishment of therapies with solid evidence-based recommendations.

**Figure 2:** Adjusted Hazard Ratio for mortality according to LVEF.

After this new HF category has been proposed, there will be increasingly more research on this type of HF, contributing to a better understanding of this new phenotype, and whether an increase in mortality for LVEF ≥65% applies to people with hypertension and obesity remains a significant question that deserves further studies.

## Conclusions

Based on the existing research, we conclude that patients diagnosed with HFsnEF (LVEF≥65%) have a special clinical manifestation, which is characterized by a higher all-cause mortality compared with other HFpEF patients.
